# The cost of implementing the Systems Analysis and Improvement Approach for a cluster randomized trial integrating HIV testing into family planning services in Mombasa County, Kenya

**DOI:** 10.1186/s12913-022-08828-z

**Published:** 2022-12-05

**Authors:** Dorothy Thomas, George Wanje, McKenna C. Eastment, R. Scott McClelland, Emily Mwaringa, Shem Patta, Walter Jaoko, John Kinuthia, Aisha Abubakar, Kenneth Sherr, Ruanne V. Barnabas

**Affiliations:** 1 Department of Global Health, University of Washington, Ninth and Jefferson Building, HMC 359927, 98104-2499 Seattle, USA; 2 Department of Medicine, University of Washington, Seattle, USA; 3 Department of Epidemiology, University of Washington, Seattle, USA; 4 Department of Medical Microbiology & Immunology, University of Nairobi, Nairobi, Kenya; 5 Department of Health Services, County Government of Mombasa, Mombasa, Kenya; 6 Department of Research & Programs, Kenyatta National Hospital, Nairobi, Kenya; 7 Department of Industrial & Systems Engineering, University of Washington, Seattle, USA; 8 Division of Infectious Diseases, Massachusetts General Hospital, Boston, USA; 9 Division of Infectious Diseases, Harvard Medical School, Boston, USA

**Keywords:** Cost analysis, HIV, Family planning, Women, Prevention, Systems analysis and improvement approach (SAIA)

## Abstract

**Background:**

Although HIV testing in family planning (FP) clinics is a promising approach for engaging women in HIV treatment and prevention services, HIV testing rates are low in FP clinics in Kenya. In 2018, a cluster randomized trial was implemented in Mombasa, Kenya applying the Systems Analysis and Improvement Approach (SAIA) to integrate HIV testing into FP services (1K24HD088229-01). We estimated the incremental costs and explored cost drivers of the FP HIV SAIA implementation in Mombasa, Kenya.

**Methods:**

We conducted a costing evaluation from the payer perspective for the FP HIV SAIA randomized control trial. We identified relevant activities for the intervention including start-up, training, research and FP HIV SAIA. We estimated activity time burden using a time-and motion study. We derived unit costs through staff interviews and programmatic budgets. We present cost estimates for two different scenarios: as-implemented including research and projected costs for a Ministry of Health-supported intervention. All costs are reported in 2018 USD.

**Results:**

For an annual program output of 36,086 HIV tests administered to new FP clients, we estimated the total annual program cost to be $91,994 with an average cost per new FP client served of $2.55. Personnel and HIV rapid testing kits comprised 55% and 21% of programmatic costs, respectively. Assuming no changes to program outputs and with efficiency gains under the MOH scenario, the estimated cost per new FP client served decreased to $1.30 with a programmatic cost reduction of 49%.

**Conclusion:**

FP HIV SAIA is a low-cost and flexible implementation strategy for facilitating integrated delivery of HIV testing alongside FP services. Although cost implications of the FP HIV SAIA intervention must continue to be evaluated over time, these findings provide context-specific cost data useful for budget planning and decision-making regarding intervention delivery and expansion.

**Trial registration:**

The trial was registered on December 15, 2016, with clinicaltrials.gov (NCT02994355).

## Background

Sub-Saharan Africa is home to over half the global population of people living with HIV [1] and reproductive age women account for an inordinate share of the global HIV burden [2–4]. There are an estimated 1.4 million Kenyan adults living with HIV—approximately 65% of whom are women [5]. In Kenya, the number of incident HIV infections observed for young women between the ages of 15 and 24 is more than double the number of new infections observed for men in the same age group [5]. In order to achieve the UNAIDS 95-95-95 fast track goals by 2030—which aim to have 95% of people living with HIV knowledgeable of their status, 95% adherent to ART and 95% virally suppressed—it is of foundational importance to amplify strategies that link people to HIV testing, prevention and treatment services [6]. While the past three decades have marked astounding accomplishments in the name of the global HIV response, there remains a startling discrepancy between the progress achieved to date and actualizing the end of this epidemic. At the end of 2020, an estimated 84% of all people living with HIV knew their status and 90% of people on treatment had suppressed viral load [7]. However, there are persistent inequalities in HIV treatment coverage—particularly in high burden, resource constrained settings—that continue to stifle progress towards the HIV response. An important threat to achieving the UNAIDS 95-95-95 goals is the failure to identify undiagnosed individuals living with HIV [8, 9]. The provision of opt-out HIV testing in family planning clinics has been found to increase receipt of HIV test results as well as the identification of new HIV diagnoses [10]. Integrating HIV testing into family planning (FP) services represents a strategic opportunity for meeting women “where they are” to ensure that they know their HIV status and are well-poised to prevent the acquisition and transmission of HIV [11]. Further, there is well-documented evidence of high acceptance and uptake of HIV testing within the context of reproductive health services and the integration of HIV testing alongside FP services is gaining traction and prioritization from global audiences [12–16].

60% of Kenyan women who access modern family planning (FP) products do so from public sector family planning clinics at government hospitals, health clinics or dispensaries and 34% of Kenyan women who access modern FP methods do so from private sector family planning clinics at private hospitals, health clinics or pharmacists [17]. 44% of women living in Mombasa County, Kenya access FP services [17]. Recent estimates from Kenya suggest that only 10% of new FP clients are offered HIV testing [18]. This is a critical missed opportunity for engaging a large proportion of reproductive age Kenyan women with substantial HIV risk. For reproductive age women, the provision of HIV testing alongside FP services is an opportunity to address reproductive health needs while concurrently offering HIV treatment and prevention services. Indeed, the World Health Organization recommends integrating HIV testing in family planning services for geographies with a high burden of HIV [19, 20]. To better serve women of reproductive age who have substantial risk of acquiring HIV or who may be living with HIV, and to promote public health on the national scale, Kenya’s National AIDS and STD Control Program encourages the provision of HIV testing and counseling at health facilities that offer FP services [21].

**Fig. 1:**
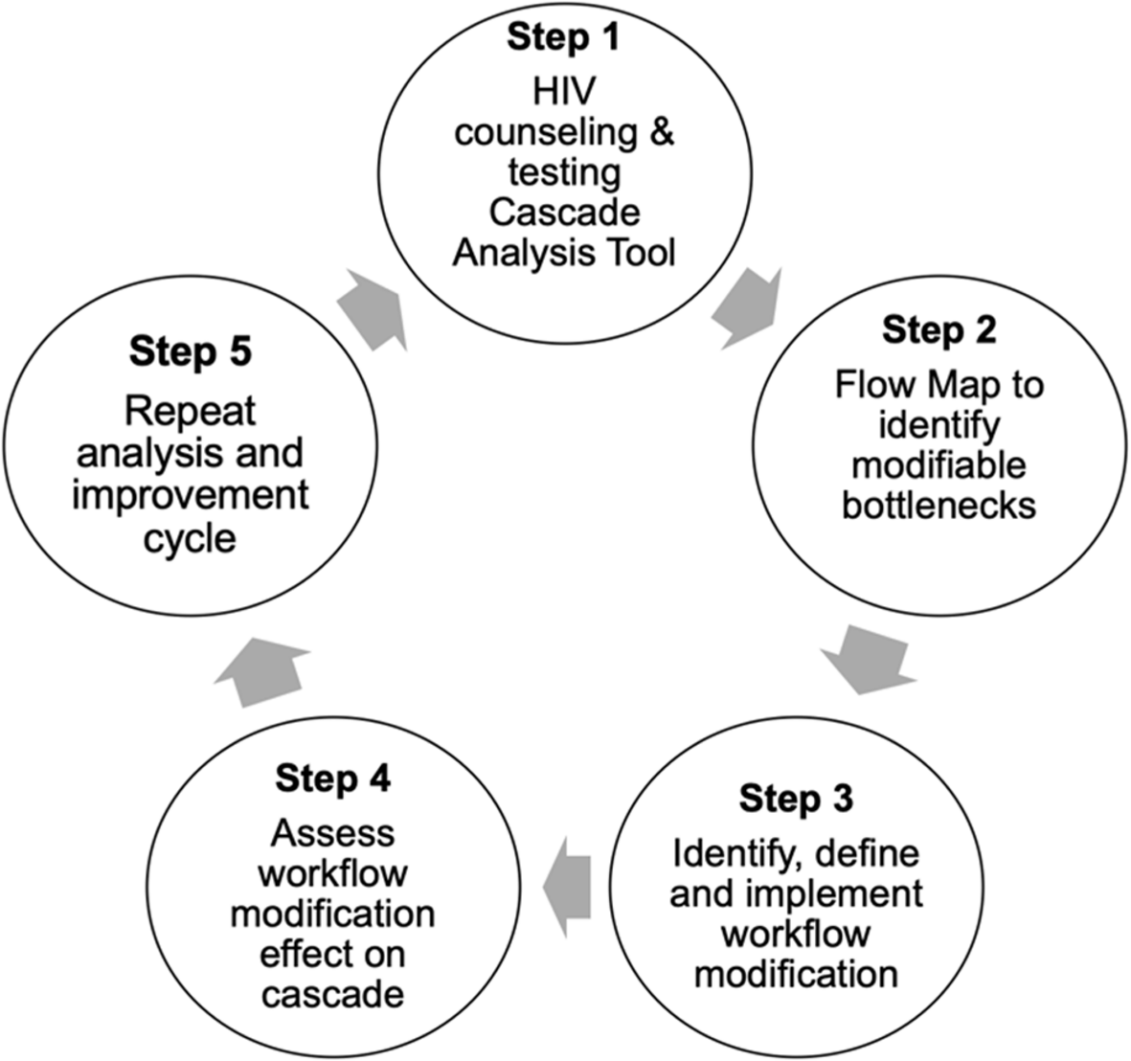
The systems analysis and improvement approach for the FP HIV SAIA implementation

In addition to supporting progress towards closing the HIV testing gap, integrating HIV testing into FP services is a promising approach for promoting improved outcomes for sexual, reproductive and maternal and child health by adapting existing health care delivery models for improved efficiency and optimization [22–25]. In 2018, a cluster randomized trial was implemented to evaluate the Systems Analysis and Improvement Approach (SAIA) for integrating HIV testing into existing FP services (FP HIV SAIA) in 12 intervention health facilities in Mombasa, Kenya (Fig. 1) [26, 27]. In a seminal cluster-randomized trial applying SAIA to the prevention of mother-to-child HIV transmission, the evidence-based strategy was found to be effective at leveraging routine programmatic data to guide decision making; supporting providers to identify and address implementation challenges; and improving HIV testing and treatment initiation [28]. FP HIV SAIA is an implementation strategy that aims to facilitate the integration of HIV testing in FP clinics by systematically assessing and managing implementation challenges as they arose [29]. Understanding the resource requirements of FP HIV SAIA has utility for decision makers interested in strategically scaling up an integrated implementation of this nature. We did not identify any literature evaluating the cost of using SAIA for integrated delivery of FP and HIV service offerings. We estimated the cost of the FP HIV SAIA implementation in Mombasa, Kenya.

## Methods

### Study setting

The FP HIV SAIA intervention was assessed via a cluster randomized trial focused on improving the implementation of existing national guidance by optimizing the effectiveness with which HIV testing is integrated into existing FP service provision. This intervention leveraged the FP HIV SAIA which aims to (1) understand and identify challenges related to integrating HIV counseling and testing in FP services (Step 1: Cascade analysis); (2) identify actionable opportunities for improvement (Step 2: Flow mapping to identify modifiable bottlenecks); and (3) test and adapt identified improvements in an iterative manner (Steps 3–5). Additional details of the SAIA approach have been outlined elsewhere [26, 27]. See Fig. 1 for more information about FP HIV SAIA.

The University of Washington closely collaborated with the Mombasa County Department of Health Services, University of Nairobi and Kenyatta National Hospital to implement the FP HIV SAIA intervention in 12 health facilities in Mombasa, Kenya, from December 2018 to November 2019. The FP HIV SAIA intervention was approved by the Kenyatta National Hospital - University of Nairobi Ethics and Research Committee and the University of Washington Human Subjects Division. Family planning clinic staff provided verbal assent to participate in this trial.

### Time and motion assessment

We estimated the time required for administering activities related to the FP HIV SAIA intervention in the 12 implementing health facilities. We conducted an observational time and motion study to describe the work activities undertaken for the FP HIV SAIA intervention in Mombasa, Kenya. We used a Microsoft Excel-based data collection instrument to collect data about work activities as well as their respective time requirements [30]. Research staff familiar with the intervention and with training and expertise in data collection observed all activities and recorded the time required for each activity. Data collection for the present analysis was conducted in all implementing health facilities in November 2019. Data collectors followed research staff and providers over the course of a typical workday and used tablets programmed with the electronic data collection software REDCap to identify the FP HIV SAIA-related activities in which they were engaged as well as the associated time dedicated to work activities [31].

Activities were assigned to four mutually exclusive categories: start-up, training, research and FP HIV SAIA. Start-up activities included initial trainings for the integrated FP HIV SAIA intervention. Training included resources required for a refresher training for the integrated FP HIV SAIA intervention that was conducted 6 months following trial initiation. Start-up and training costs were derived from programmatic expenditure reports. Research activities included image upload to the research database, research database management and conducting calls to coordinate FP HIV SAIA visits with FP facility staff. FP HIV SAIA activities included transportation to facilities for FP HIV SAIA, providing supportive supervision for FP HIV SAIA to facility staff and abstracting data from the FP register for the FP Cascade Analysis tool (i.e., Documentation outlining Step 1 of SAIA) and for outcomes assessment. Data collectors indicated the duration of each activity using the start and end time for each task. Our time and motion analysis leveraged task durations to calculate the total amount of time engaged in each activity. To further summarize time and motion observations, we estimated the mean time spent on each activity. To estimate personnel costs for each observed activity, we multiplied mean time estimates by the associated hourly personnel cost.

### Costing

We followed principles outlined in the Global Health Cost Consortium Reference Case to conduct an activity based micro-costing assessment from the payer perspective [32]. In addition to the time and motion assessment, costing data were supplemented by available literature on public and private health salaries in Kenya, interviews with research staff, interviews with local experts to verify cost estimates, and reviews of study budgets and invoices (Table 1). Start-up costs were annualized assuming a useful life of 5 years. Taking into consideration national holidays, weekends and sick leave, we estimated that facility staff worked 219 days annually and 7 h daily. Monthly health facility personnel costs were derived using a mean estimate (informed by expert consultation and literature on Kenya-specific earnings for nurses) of gross monthly salary for intermediate grade nurses from public and private facilities [33]. Hourly personnel costs were derived by dividing monthly salary by the number of hours worked per month on SAIA (See Supplement for summary of staffing wage assumptions). Capital costs were obtained from the study budget and published estimates. The costs of HIV rapid test kits were obtained from the Global Fund to Fight AIDS, Tuberculosis and Malaria [34]. Costs are reported in 2018 USD. To convert currency from Kenyan Shillings to USD, we used an OANDA exchange rate of 101.95 KSh per 1 USD [35].

**Table 1:** Unit costs of key FP HIV SAIA components

Item	Cost (2018 USD)	Source
Middle tier civil service nurse monthly salary + allowances	1406.80	MOH Salary Scale
Private sector nurse monthly salary + allowances	750.37	Evaluation of public/private wage differences in Kenya (33) Study personnel
Lower tier civil service nurse monthly salary + allowances	611.26	MOH Salary Scale
Lead researcher monthly salary + allowances	2323.68	Study budget
Senior research clinical officer monthly salary + allowances	619.12	Study budget
Research nurse monthly salary + allowances	600.48	Study budget
Research assistant monthly salary + allowances	556.34	Study budget

Data analysis was conducted using Microsoft Excel 2018. We estimated costs for two scenarios: (1) As-implemented costs including the research components; (2) FP HIV SAIA as theoretically implemented by the Ministry of Health.

### Scenario 1: As-implemented costs including the research components

This scenario represents the costs associated with carrying out this implementation in the 12 intervention facilities. Personnel costs include the monthly gross salaries for associated clinical research or facility staff responsible for carrying out each activity. In this scenario, research staff worked in teams of two to carry out research and FP HIV SAIA activities. Start-up costs include a full day of offsite training led by research project staff for health facility staff involved in the integrated FP HIV SAIA intervention. Refresher training costs include those resources required for a half-day off-site training led by research project staff for health facility staff involved in the intervention. Research costs include resources required for research staff to upload data to the research database, manage the research database and coordinate with facility staff for FP HIV SAIA visits.

### Scenario 2: SAIA as theoretically implemented by the County Ministry of Health

This scenario is reflective of the estimated implementation costs if implemented by the Mombasa County Department of Health Services in 140 public and private family planning clinics in Mombasa. These health facilities are routinely staffed by clinical officers and nurses. To obtain personnel costs for this scenario, we revised the pay structure to be reflective of a lower tier (i.e., Grade H) public sector nurse (Table 1). Personnel costs include the monthly gross salaries for Grade H public sector nurses. Start-up costs include one half-day off-site training led by two clinical officers and two senior nurses. In this scenario, the length of the training was revised to reflect the exclusion of research-related components. Finally, time allocated to data abstraction was reduced to 34% of the observed time in an effort to reflect an anticipated reduction in time-burden when implemented in a non-research environment (i.e., abstracting data from the FP register for the Family Planning Cascade Analysis Tool).

## Results

### Time and motion

In the 12 intervention facilities, research staff spent an average of 19 min per month providing supportive supervision to staff at intervention facilities (e.g., discussing the Family Planning Cascade Analysis Tool), 120 min in round trip transportation to intervention facilities, 74 min uploading images and 5 min synching data to the online research database. Facility staff spent an average of 23 min abstracting data from FP register and 4 min coordinating with research staff for SAIA visits (Table 2). A total of 21 facility staff were observed during data collection visits, an average of 1.8 staff per implementing facility. For the MOH scenario, we applied an estimated 34% of the observed time for abstracting data from FP register for the cascade analysis tool as routine components of FP HIV SAIA.

**Table 2:** Time and motion estimates (minutes) for FP HIV SAIA delivery in intervention facilities

	Intervention Facility
**Activity**	**Average time (minutes)**
*FP HIV SAIA*	
Supervision	18.8
Transportation	120
Image upload	73.8
Database management	5
Data abstraction	23.3
Telephone follow-up	4.3

### Overall program costs and unit costs

The 12 intervention facilities saw 10,852 total family planning clients—3093 of whom were new clients. There was a median number of 262 (Interquartile range [IQR]: 135–361) new and 637 (IQR: 496–1286) total and FP clients seen per facility during the study period.

For an annual program output of 36,085 HIV tests administered to new FP clients, the estimated total annual program cost was $91,994 for the As-Implemented Scenario. The estimated mean cost per new FP client served was $2.55. As indicated in Tables 3 and 4, when considering the four main activity categories, FP HIV SAIA presented the greatest burden to programmatic cost (52.5%), followed by HIV testing kits (31.7%), research (12%), start-up (3%) and refresher training (< 1%) costs. Across all cost categories, personnel was the most substantial cost driver (55%) followed by HIV testing kits (31.8%). The allocation of personnel costs across the different cost categories is outlined in Tables 3 and 4.

**Table 3:** Facility level monthly cost (2018 USD) of delivering FP HIV SAIA intervention

	Scenario 1	Scenario 2
**Number of facilities**	12	140
**Number of new clients per facility**	22	22
**START UP**		
Trainers (Personnel)	0.27	0.01
Travel reimbursement	0.26	0.08
Refreshments	0.29	0.03
Printing	0.82	0.21
* Total Monthly Start-up Costs*	*1.64*	*0.33*
**REFRESHER TRAINING**		
Trainers (Personnel)	0.15	0
Travel reimbursement	0.19	0
Refreshments	0.07	0
* Total Monthly Refresher Costs*	*0.41*	*0*
**RESEARCH**		
Image upload (Personnel)	5.35	0
Database management (Personnel)	0.73	0
Telephone follow-up (Personnel)	0.31	0
Airtime	0.17	0
* Total Monthly Research Costs*	*6.56*	*0*
**FP HIV SAIA**		
Supervision (Personnel)	2.73	2.02
Transportation (Personnel)	17.4	4.85
Data abstraction (Personnel)	3.39	0.65
Public transportation fees	5.23	2.62
* Total Monthly FP HIV SAIA Costs*	*28.75*	*10.14*
* Total Monthly HIV Testing Kit Costs*	*17.39*	*17.39*
* Total Monthly Costs Per Facility*	*54.76*	*27.87*
* Total Monthly Costs Per New Client*	2.55	1.30

**Table 4:** Total annual cost (2018 USD) of delivering FP HIV SAIA intervention

	Scenario 1	Scenario 2
**Number of facilities**	140	140
**Total number of tests administered to new clients**	36,085	36,085
**START UP**		
Trainers (Personnel)	453.6	16.8
Travel reimbursement	436.8	134.4
Refreshments	487.2	50.4
Printing	1377.6	352.8
* Total Annual Start-up Costs*	*2755.2*	*554.4*
**REFRESHER TRAINING**		
Trainers (Personnel)	252	0
Travel reimbursement	319.2	0
Refreshments	117.6	0
* Total Annual Refresher Costs*	*688.8*	*0*
**RESEARCH**		
Image upload (Personnel)	8988	0
Database management (Personnel)	1226.4	0
Telephone follow-up (Personnel)	520.8	0
Airtime	285.6	0
* Total Annual Research Costs*	*11,020.8*	*0*
**FP HIV SAIA**		
Supervision (Personnel)	4586.4	3393.6
Transportation (Personnel)	29,232	8148
Data abstraction (Personnel)	5695.2	1092
Public transportation fees	8786.4	4401.6
* Total Annual FP HIV SAIA Costs*	*48,300*	*17,035.2*
* Total Annual HIV Testing Kit Costs*	*29,228.85*	*29,228.85*
* Total Annual Costs*	*91,993.65*	*46,818.45*
* Total Annual Costs Per Facility*	*657.10*	*334.42*
* Total Annual Costs Per New Client*	*2.55*	*1.30*

### Projected costs under Ministry of Health scenario

We estimated costs under the Ministry of Health (MOH) scenario in which we assumed stable implementation outputs. In this scenario, we substituted lower-tiered (i.e., Grade H) public sector clinical staff salaries for the combined private/public intermediate-tiered salaries applied in Scenario 1. We have excluded research and refresher training costs from this scenario. To characterize anticipated changes in implementation quality, cost projections for the MOH scenario were reduced and represent 34% of Scenario 1 data abstraction time. In the MOH scenario, the estimated total annual program cost was $46,819 and the average cost per new FP client served was $1.30, which represents a 49% reduction in costs in comparison to Scenario 1 (Tables 3 and 4). HIV testing kits represented the largest cost burden (62.4%), followed by FP HIV SAIA (36.4%) and start-up (1.2%). Across all cost categories, personnel comprised 27% of annualized total program costs for the MOH scenario which represents a 75% cost reduction from estimated personnel costs in Scenario 1.

We conducted a sensitivity analysis to evaluate changes to program costs when applying different assumptions regarding program delivery for the Ministry of Health FP HIV SAIA implementation. Namely, we considered the influence of changing the estimated amount of time allocated to data abstraction so that it reflected 60% and 120% of the observed data abstraction time. Increasing the estimated amount of time for data abstraction from 34 to 60% of what was observed in Scenario 1 increased the cost per new client served to $1.32 while increasing the total program costs by 2%. Increasing data abstraction time to be 120% of what was observed in Scenario 1 increased the cost per new client served to $1.37 while increasing the total program costs by 6%. Additionally, we assessed changes to program costs when halving and doubling the number of new FP clients served (Table 5). Halving the number of new FP clients (from 36,085 to 18,043 clients) served increased the cost per client served to $1.78 and reduced annual program costs by 31%. By contrast, doubling the number of new FP clients (from 36,085 to 72,170 clients) served decreased the cost per client served to $1.05 and increased annual program costs by 62%. See Table 5 for additional details of sensitivity analyses.

**Table 5:** Sensitivity analysis results for Ministry of Health implemented FP HIV SAIA (2018 USD).

Scenario	Total annual cost	Cost per new client served
Ministry of Health implemented	46,818.45	1.30
60% Data abstraction time	47,623.45	1.32
120% Data abstraction time	49,520.45	1.37
Halving new clients served	32,204.43	1.78
Doubling new clients served	76,047.30	1.05

## Discussion

We used primary data from the intervention arm of a trial testing the efficacy of FP HIV SAIA for integrating HIV testing into existing family planning services in Mombasa, Kenya. To our knowledge, this evaluation is the first to outline the cost of SAIA for integrated FP HIV. We estimated the costs associated with the implementation of an intervention intended to improve the effectiveness with which HIV testing is delivered alongside standard FP services. We estimated that the annual cost of FP HIV SAIA as performed in the research as-implemented scenario was, on average, $2.55 per new FP client served. Personnel and HIV testing kits accounted for 55% and 32% of estimated programmatic costs, respectively. Overall program costs decreased by 49% under the MOH implementation relative to the as-implemented scenario. Implementation studies such as this are essential for understanding the cost implications of delivery models that aim to optimize the introduction of HIV testing within the context of FP clinics. These findings provide valuable insights regarding the resource requirements for the implementation and potential scale up of FP HIV SAIA in Kenya and similar contexts.

Our assessment identifies FP HIV SAIA as a low-cost approach for optimizing the introduction of HIV testing in existing FP services. Similar to an evaluation of a SAIA implementation for the prevention of mother-to-child HIV transmission, we found FP HIV SAIA to be suitable for application in limited resource settings with this low-cost and contextually grounded approach showing promise for sustained service delivery improvements [28, 36]. The co-location of HIV testing services alongside existing health offerings has demonstrated effectiveness for improving the uptake of HIV testing in diverse settings [25, 37]. Given the dearth of costing data specific to the application of SAIA for integrating HIV testing alongside FP services, we contextualize our study findings as a percentage of the incremental cost of integrating HIV testing into FP services. Cost assessments of integrating HIV testing into public health facilities in Malawi, Zambia and Zimbabwe provide estimates between $4.24 and $8.79 for each client that tested for HIV [38]. In a cost evaluation in Kenyan public health facilities, the incremental cost of integrating HIV testing into FP services was estimated to be $5.60 per client served [25, 39]. Our FP HIV SAIA valuation of $2.55 per new FP client served represents 45.5% of this estimate. Notably, our estimate is specific to the number of new clients served whereas our counterparts establish incremental cost estimates for the total number of clients served. This discrepancy reflects a greater programmatic volume in the Liambila et al. study relative to our own that, if better matched across the two evaluations, would further suppress the proportionate cost burden of FP HIV SAIA. It is important to highlight the overall low cost of implementing SAIA and reiterate that HIV testing kit costs account for a substantial share of the cost burden. Further, despite limited SAIA-specific cost data, assessments of integrated delivery of pre-exposure prophylaxis and ART have demonstrated effectiveness for preventing incident cases of HIV and have identified personnel and medication as key cost drivers—with HIV testing representing a minor burden on incremental costs for the integrated program [40–44]. As the integration of HIV testing with existing health services continues to gain traction, it will be important to continue evaluating programmatic performance in order to identify opportunities for improving service delivery. A systematic review of integrated FP and HIV services identified addressing underlying health systems challenges as critical for potential FP HIV testing integration and optimization efforts [45]. SAIA is well-positioned to address this threat to FP HIV integration because it concurrently promotes implementation of evidence-based strategies while leveraging an iterative approach to identify and address context-specific systems-level implementation challenges.

There are limitations and considerations that are useful for further contextualizing study findings. Projected costs under the MOH scenario are sensitive to assumptions about program volume. Instances of HIV testing kits and/or FP commodity stockouts would also negatively influence programmatic output. Unit costs are similarly sensitive to program output as well as changes to staff and supervisory structures. Of note, non-research staff may be less efficient at implementing FP HIV SAIA. With more robust community awareness and sensitization, uptake of HIV testing may increase over time in FP and other health settings. We did not address whether additional staff would be required to support the adoption of HIV testing into routine FP services. If more staff are required for widescale programmatic success, this might necessitate additional human resources or the provision of different cadres of health workers such as HIV peers or counsellors. Potential impacts of changing the human resource allocation for integrated FP HIV services include increased personnel costs; reduced quality of FP service delivery; and reduced provider and client satisfaction. An additional monitoring concern (and potential focus for FP HIV SAIA cycles) for integrating HIV testing into FP service offerings will be ensuring that key information regarding HIV testing is appropriately documented and consolidated into relevant HIV information management systems. The application of FP HIV SAIA will likely be time-limited and will, thus, require that implementers identify the most appropriate timeline for leveraging this implementation strategy for optimized integration. Relatedly, the acceptability of HIV testing alongside FP services has important implications for programmatic usership and, resultingly, programmatic cost estimates. Under the MOH scenario, it is possible that the intervention may be less efficiently administered and/or or have lower effectiveness when delivered by staff who are unsupported by dedicated researchers. The as-implemented scenario may provide insights into the upper bounds of potential costs incurred when delivered within the context of a less efficient system. The facilities included in this intervention (and the related estimated programmatic costs) may not be representative of all FP clinics throughout Kenya. Despite efforts to ensure the comfort of observed staff and assure them that their participation in this research would be used for research purposes only, the Hawthorne effect represents an additional potential methodological limitation. As the evidence base outlining the cost of leveraging SAIA for HIV testing integration grows so, too, will our confidence in generalizing these estimates to external settings. Despite the aforementioned considerations, the reported cost estimates are important instruments for assessing the relative cost and budget impact of introducing FP HIV SAIA to existing programmatic offerings. These estimates will be useful for establishing benchmarks against which findings from other SAIA cost evaluations may be compared. Notable benefits associated with integrating HIV testing into existing FP services include promoting improved HIV testing access to a priority population as well as programmatic efficiency. The joint provision of HIV testing in FP clinics may promote increased programmatic efficiency by leveraging economies of scope. Furthermore, these findings are timely and relevant given the high HIV burden among Kenyan reproductive age women as well as the World Health Organization and the Ministry of Health Kenya National AIDS and STD Control Program’s support of offering HIV testing alongside FP services. Better understanding the cost implications of leveraging the SAIA approach as tool for integrating HIV testing into existing FP service offerings represents an important public health investment towards better serving affected populations for HIV prevention and treatment in Kenya and similar limited-resource settings.

## Conclusion

The Systems Analysis and Improvement Approach is an evidence-based implementation strategy that can be used for facilitating the integration of HIV testing into existing family planning services. Leveraging MOH payment structures and limiting the amount of time spent abstracting client data has the potential to reduce costs from the As-Implemented research scenario. Further, reducing the number of new clients served—for example through targeted testing strategies and/or a natural easing of the volume of new clients served—also has the potential to reduce overall program costs. With increasing interest in and adoption of SAIA, it will be critical to continue evaluating the cost and programmatic implications of FP HIV SAIA particularly in geographies and amongst populations with a high HIV burden. These findings may be used for the continued evaluation of cost effectiveness and efficiency of SAIA. Additional contextually grounded evaluations of FP HIV SAIA will be needed to inform its adoption, adaptation and scale-up.

## Data Availability

The data used for the present study are available from the corresponding author upon reasonable request.

## Abbreviations

ART: Antiretroviral therapy
FP: Family planning
HIV: Human immunodeficiency virus
KSh: Kenyan Shilling
MOH: Ministry of Health
SAIA: Systems Analysis and Improvement Approach
STD: Sexually Transmitted Disease
UNAIDS: Joint United Nations Programme on HIV/AIDS
USD: United States dollar
